# The Dual Roles of Triiodothyronine in Regulating the Morphology of Hair Cells and Supporting Cells during Critical Periods of Mouse Cochlear Development

**DOI:** 10.3390/ijms24054559

**Published:** 2023-02-25

**Authors:** Xue Bai, Kai Xu, Le Xie, Yue Qiu, Sen Chen, Yu Sun

**Affiliations:** 1 Department of Otorhinolaryngology, Union Hospital, Tongji Medical College, Huazhong University of Science and Technology, Wuhan 430022, China; 2 Hubei Province Key Laboratory of Oral and Maxillofacial Development and Regeneration, Wuhan 430022, China

**Keywords:** triiodothyronine, hearing loss, cochlear remodeling, organ of corti

## Abstract

Clinically, thyroid-related diseases such as endemic iodine deficiency and congenital hypothyroidism are associated with hearing loss, suggesting that thyroid hormones are essential for the development of normal hearing. Triiodothyronine (T3) is the main active form of thyroid hormone and its effect on the remodeling of the organ of Corti remain unclear. This study aims to explore the effect and mechanism of T3 on the remodeling of the organ of Corti and supporting cells development during early development. In this study, mice treated with T3 at postnatal (P) day 0 or P1 showed severe hearing loss with disordered stereocilia of the outer hair cells (OHCs) and impaired function of mechanoelectrical transduction of OHCs. In addition, we found that treatment with T3 at P0 or P1 resulted in the overproduction of Deiter-like cells. Compared with the control group, the transcription levels of Sox2 and notch pathway-related genes in the cochlea of the T3 group were significantly downregulated. Furthermore, Sox2-haploinsufficient mice treated with T3 not only showed excess numbers of Deiter-like cells but also a large number of ectopic outer pillar cells (OPCs). Our study provides new evidence for the dual roles of T3 in regulating both hair cells and supporting cell development, suggesting that it is possible to increase the reserve of supporting cells.

## 1. Introduction

The organ of Corti (OC), the auditory sensor by which sounds are converted into nerve impulses, is one of the most complex organ structures in mammals. The structure includes one row of inner hair cells (IHCs) and three rows of outer hair cells (OHCs) interdigitated among different types of supporting cells (SCs). The morphology of different SCs is distinct and few studies have focused on their biology and function [1]. Abnormal development of SCs induced by gene mutations, congenital cytomegalovirus infection, or thyroid disease can lead to severe hearing loss [2,3,4]. In addition, growing evidence suggests that well-formed SCs act as mediators of hair cell development, function, and survival. Therefore, further research on the proliferation, maturation, and development of SCs may increase our understanding of inner ear development and regeneration.

In rodents, the overall structure of the auditory epithelium is formed by the time of birth and continues to mature structurally until hearing begins on postnatal day (P)14. During this period, HCs form V-shape stereocilia that accommodate the mechanoelectrical transduction (MET) channels. Deiter cells (DCs), a type of SCs coupled with OHCs, extend phalangeal processes to provide structural support for OHCs. Inner and outer pillar cells (IPCs and OPCs) form triangular tunnels of Corti which support the whole OC. More different types of SCs, such as Hensen’s cells, Claudius cells, or inner sulcus cells, lie lateral to OHCs or IHCs. Many previous studies have focused on the molecules that regulate the proliferation and differentiation of hair cells (HCs) in order to achieve the purpose of restoring impaired hearing through HC regeneration strategies [5,6,7]. Although ectopic HCs can be induced in the cochleae of newborn or adult mice, due to the lack of corresponding SCs and fine OC structure, these efforts still failed to restore hearing and maintain it in the long term [8,9,10]. For the treatment of deafness, reconstruction of the entire OC may be a better but more difficult choice. It is particularly important to explore the development and regeneration of SCs. Interestingly, SC development is regulated by both local molecular pathways and systemic hormones.

The thyroid hormone regulates OC formation and the development of normal hearing [11,12]. Triiodothyronine (T3) is the main active form of thyroid hormone and acts on thyroid hormone receptors (TRs) to induce a series of physiological changes in target tissues [13,14]. In particular, animals with developmental hypothyroidism exhibit delayed opening of the tunnel of Corti [15,16]. Conversely, excess T3 can lead to the death of SCs in the greater epithelial ridge (GER) and early opening of the tunnel of Corti with premature PCs [17,18]. These findings suggest that T3 regulates the development of some types of SCs in the cochlea. In humans, thyroid-related diseases such as endemic iodine deficiency, congenital hypothyroidism, and resistance to T3 caused by mutations in TRs are associated with hearing loss [19,20,21]. In mice, secondary hypothyroidism caused by mutations in TRs results in permanent potassium channel dysfunction and impaired HC function [22,23,24]. Knockout of T3 transporters can lead to OHC death and developmental arrest of SCs in mice [25]. These findings suggest that T3 plays multiple roles in the function of HCs and the development of SCs, especially in the fine structure formation of the OC.

To explore the effect of T3 on the remodeling of the OC and the development of SCs during early development, we administered T3 to neonatal mice at different time points after birth. Our results show that excess T3 given at an early stage (P0 or P1) leads to severe hearing loss with abnormal stereocilia alignment and HC mechanosensory dysfunction. Moreover, mice in the P0 or P1 group showed an overproduction of Deiter-like cells. These extra cells expressed the functional marker acetylated α-tubulin and are linked to adjacent DCs through gap junctions. A series of genes related to the development of cochlear sensory epithelium were significantly downregulated in the T3 group. When Sox2 haploinsufficient mice were treated with T3 at P0, the number of DCs and OPCs increased significantly and resulted in a great change in the structure of the OC. Our finding suggests that excess T3 may lead to deafness by interfering with the normal stereocilia formation and amplification function of HCs. Excessive T3 or T3 combined with Sox2 downregulation can alter the fine structure of the OC by regulating the development of SCs. Hormone action combined with key signaling pathways in the inner ear may be a potential research focus for regulating OC development and regeneration.

## 2. Results

### 2.1. Excess T3 in the Early Postnatal Days Can Induce Hearing Loss in Mice

**Figure 1:**
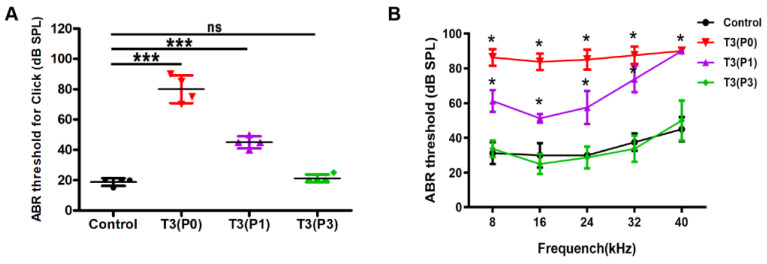
Excessive T3 in the early postnatal days can induce hearing loss in mice. (**A**) ABR-click thresholds in control and different T3-treated groups. (**B**) Comparison of tone-burst thresholds in different groups. *n* = 4 mice in each group. Data are presented as means ± SD, each experimental group vs. control. ns: not significant, * *p* < 0.05, *** *p* < 0.001.

To evaluate the effect of T3 treatments at different postnatal periods on auditory function in mice, ABR testing was performed on mice in the control group and T3 treatment group (*n* = 4 mice in each group) at P18. Compared with the control group, the ABR-click thresholds increased significantly in the P0 or P1 group, while mice in the P3 group showed normal hearing (Figure 1A). Mice in the P0 group showed severe deafness with mean thresholds above 80 dB SPL at 8–40 kHz, while mice in the P1 group displayed moderate to severe deafness with hearing thresholds at 8, 16, 24, 32, and 40 kHz of 61.3 ± 6.3, 51.3 ± 2.5, 57.5 ± 9.6, 73.8 ± 7.5, and 90.0 ± 0 dB SPL, respectively (Figure 1B). Mice treated with T3 at P3 showed normal hearing at P18 (Figure 1B).

### 2.2. Excess T3 Accelerates the Maturation of the GER and Does Not Affect HC Survival

**Figure 2:**
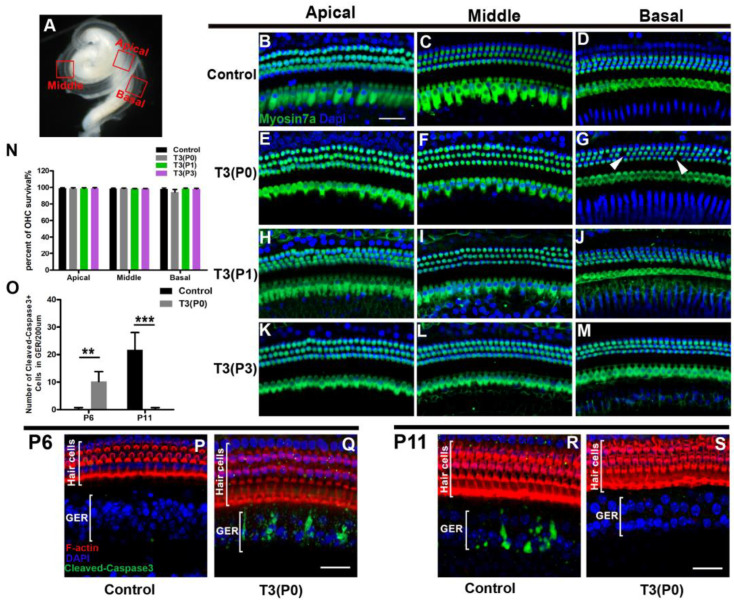
Excessive T3 accelerates the maturation of GER. (**A**) An overall image of the cochlea to show the different turns of the basilar membrane. (**B**–**M**) Representative confocal images of HCs (Myosin7a, green) of apical, middle, and basal turns in different groups at P18. White arrowheads indicate sporadic missing OHCs (**G**). (**N**) Quantifications of OHC survival in different groups at P18. (**P**,**Q**) Representative images of caspase3^+^ cells (green) in GER of the apical turn of the control group or P0 T3 group at P6. (**R**,**S**) Representative images of caspase3^+^ cells in GER of apical turns of the control group or P0 T3 group at P11. (**O**) Quantifications of caspase3^+^ cells of GER in the apical turn of control or P0 T3 groups at P6 and P11. Data are presented as means ± SD. ** *p* < 0.01, *** *p* < 0.001). Scale bar: 40 μm (**B**,**R**).

HC loss is a major cause of hearing loss. Thus, we analyzed the survival patterns of HCs in T3-treated mice. No substantial HC loss was observed in the different T3-treated groups at P18 (Figure 2B–M). Although scattered losses of OHCs were occasionally observed in the basal turn of the P0 group (white arrows, Figure 2G), statistical analysis showed that the number of OHCs was not significantly changed (*n* = 4/group, *p* > 0.05) (Figure 2N).

In neonatal mice, the cochlea continues to develop structurally and functionally before hearing onset, and regression of the GER is a prominent event. During natural development, cells in the GER promote the development and maturation of sensory epithelium through programmed cell death. We performed activated caspase-3 staining to determine the apoptosis pattern of the GER in T3 P0 treated mice. At P6, no activated caspase-3-positive (caspase-3+) cells were detected in the GER of control mice, while a large number of caspase-3+ cells were observed in the GER of the T3 P0 treated group (Figure 2P,Q). In contrast, caspase-3+ cells were evident in the GER of control cochleae and were not detected in the T3 P0 treated group at P11 (Figure 2R,S). Statistical analysis showed that the number of caspase-3+ cells differed significantly between the two groups at P6 and P11 (*n* = 4, *p* < 0.01) (Figure 2O).

### 2.3. Excess T3 Interferes with the Morphology of OHC Stereocilia and the Function of the MET Channel

**Figure 3:**
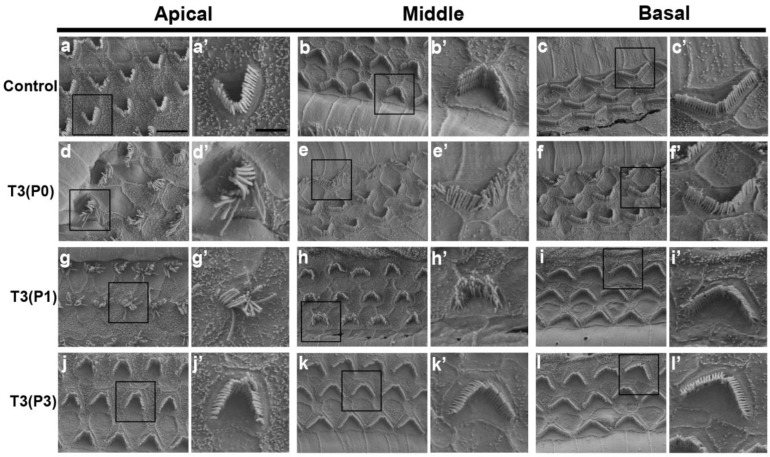
Excessive T3 interferes with the morphology of OHCs stereocilia. (**a**–**c**) The morphology of OHCs stereocilia in different turns of the control group. (**a’**–**c’**) Magnifications of the stereocilia in different turns of the control group. (**d**–**l**) Representative images of OHCs stereocilia in the apical, middle, and basal turns of different T3 treated groups. (**d’**–**l’**) Magnified images show the morphology of the stereocilia in different groups. *n* = 4 mice in each group. Scale bar: 20 μm (**a**),10μm (**a’**).

In mammals, stereocilia are located in the cuticular plate of HCs and are responsible for converting mechanical vibrations generated by sound stimulation into electrical signals. Structural or functional defects of the stereocilia are one of the main causes of congenital or progressive deafness. We performed SEM to characterize the morphology of stereocilia in different T3-treated groups. In the control group, three rows of stereocilia formed V-shaped bundles in all turns (Figure 3a–c,a’–c’). However, stereocilia bundles of OHCs in the apical and middle turns of the P0 or P1 group were disordered and lost their V-shaped structure. Interestingly, there were no obvious changes in the morphology of the stereocilia bundles in the basal turn of the P0 or P1 groups (Figure 3d–i,d’–i’). In contrast, the morphology and arrangement of the OHC stereocilia bundles were almost unaffected when T3 was given at P3 (Figure 3j–l,j’–l’). These results suggest that the abnormal arrangement of the OHC stereocilia bundle may be strongly associated with hearing loss caused by excess T3.

**Figure 4:**
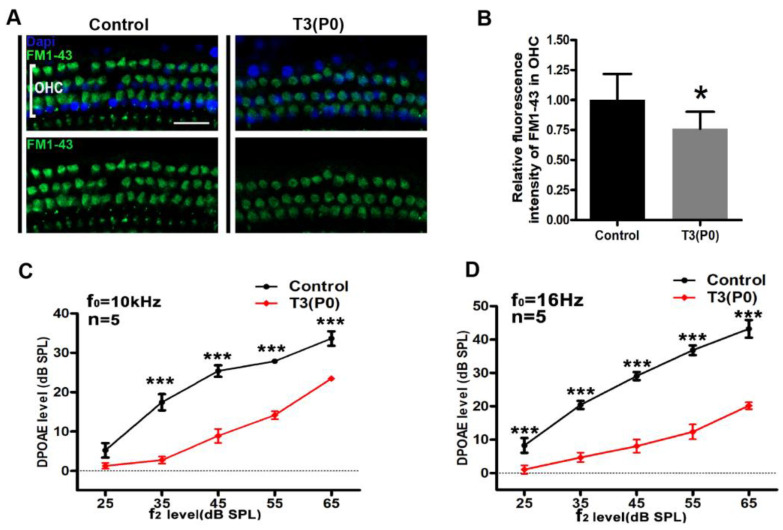
Excessive T3 interferes the function of the MET channel of OHCs. (**A**) Representative images of FM1-43 uptake by OHCs in the control and the P0 group. (**B**) Quantification of FM1-43 fluorescence in OHCs in different groups. (**C**) The DPOAE input/output plots at 10 kHz. (**D**) The DPOAE input/output plots at 16 kHz. *n* = 4 mice in each group, Data are presented as means ± SD, each experimental group vs. control. ns: not significant, * *p* < 0.05, *** *p* < 0.001.* Significantly different from control group (* *p* < 0.05, *** *p* < 0.001). Scale bar: 40 μm (**A**).

In addition, FM1-43 loading of OHCs was used to assess the function of the MET channel. Compared with the control group, the uptake of FM1-43 by OHCs in the T3 treatment group was reduced (Figure 4A). Quantitative results showed that the relative fluorescence density of FM1-43 in OHCs of T3-treated mice decreased by 23.9 ± 13.9% (Figure 3B). These results indicated that abnormalities of the HC stereocilia bundles and dysfunctions of the MET channel might be responsible for the hearing loss induced by excess T3. At both 10 and 16 kHz, the DPOAE input/output plots measured from the P0 group decreased significantly compared with the control group (Figure 3C,D). The level of DPOAE in the P0 group was significantly lower than that in the control group at all input levels (*p* < 0.001, *n* = 5 in each group).

### 2.4. Excess T3 Induces Overproduction of Deiter-like Cells 

**Figure 5:**
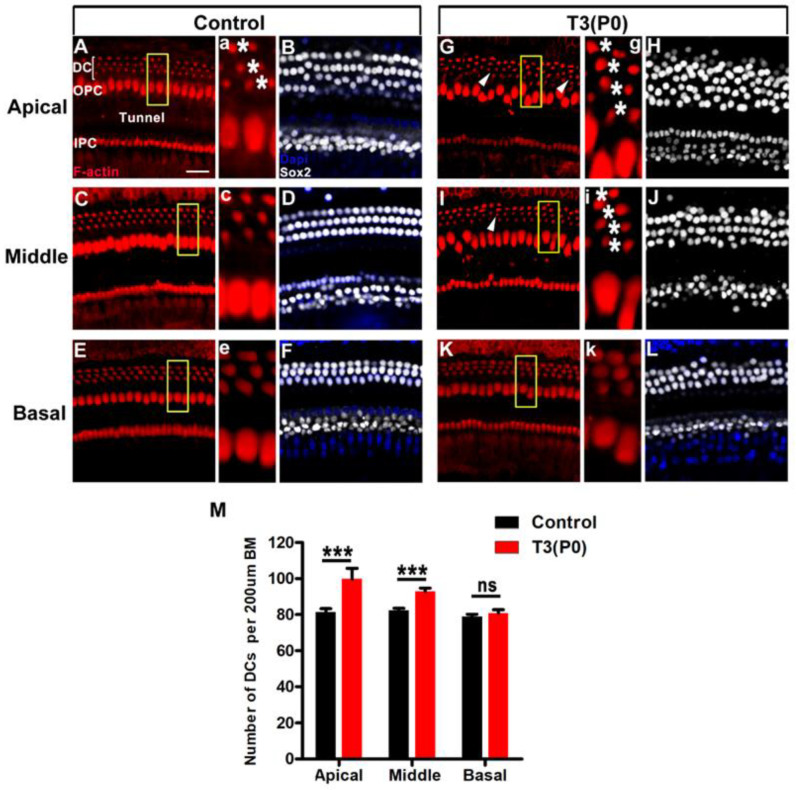
Excessive T3 can induce overproduction of DCs. (**A**,**C**,**E**) Representative images of DCs (F-actin, red) of different turns in the control group at P18. (**a**,**c**,**e**) Magnified images of yellow boxes in the panel **A**, **C**, and **E**. * indicates the bottom of a DC. (**B**,**D**,**F**) Representative images of SCs (Sox2, white) of different turns in the control group at P18. (**G**,**I**,**K**) Representative images of DCs (F-actin, red) of apical, middle, and basal turns in the P0 group at P18. White arrowheads indicate extra DCs in the P0 group (**G**,**I**). (**g**,**i**,**k**) Magnified images of yellow boxes in the panel **G**, **I**, and **K**. * indicates that DCs are arranged in four rows in the apical and middle turn of the P0 group (**g**,**i**). (**H**,**J**,**L**) Representative images of SCs of different turns in the P0group at P18. (**M**) Comparison of the number of DCs at specific cochlear locations in control and T3 treatment groups. Data are presented as means ± SD, ns: not significant, *** *p* < 0.001. Scale bar: 40 μm (**A**).

To investigate the effect of T3 on OC remodeling, mice were sacrificed at P18 and the SCs were labeled with Sox2 (white). Furthermore, phalloidin (red) was used to label the bases of the DCs and PCs. In the control group, the DCs were neatly arranged in three rows and the PCs were arranged in a single row in all turns (Figure 5A–F). However, in the P0 group, we observed four rows of DCs in the apical turns, and sporadic extra DCs in middle turns, indicating the production of extra Deiter-like cells. In addition, the arrangement of Sox2-labeled SCs was disordered and the OPCs were disordered compared to the control group (Figure 5G–J). The arrangement of DCs in the basal turn was almost unaffected in the P0 group (Figure 5K,L). Statistical analysis showed that the number of DCs (including Deiter-like cells) was significantly increased in the apical and middle turns (*n* = 4, *p* < 0.001) (Figure 5M). 

**Figure 6:**
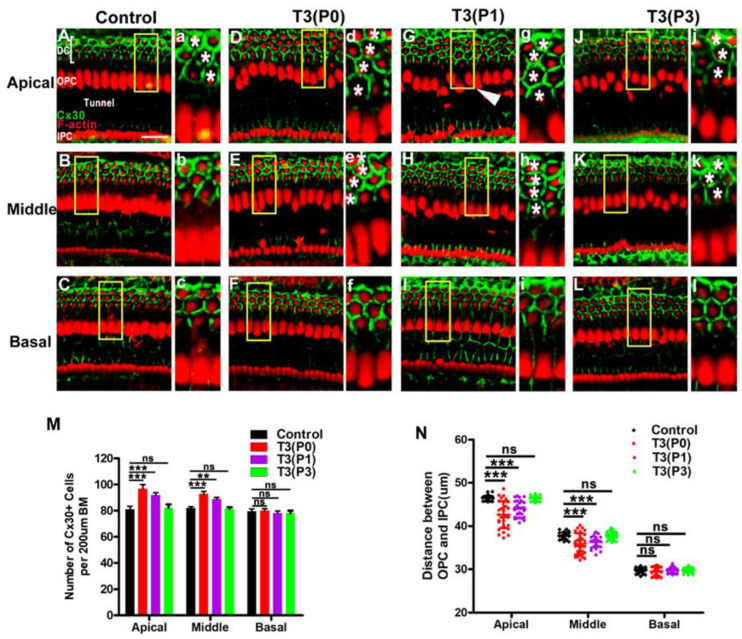
Extra DCs can be only induced by T3 in the P0 or P1. (**A**–**C**) F-actin (red) and Cx30 immunolabeling (green) in different turns of the control group, respectively. (**a**–**c**) Magnified images of yellow boxes in panel **A**–**C**. Asterisk indicates DCs were arranged in three rows in the control group (**a**). (**D**–**F**) Representative images of DCs and Cx30 expression patterns of different turns in the P0 group, respectively. (**d**–**f**) Magnified images of yellow boxes in panel **D**–**F**. The asterisk indicates the region in that DCs were arranged in four rows in the apical and middle turns (**d**,**e**). (**G**–**I**) Representative images of DCs and Cx30 expression patterns of different turns in the P1 group, white arrowheads indicate extra DCs in the P1 group. (**g**–**i**) Magnified images of yellow boxes in panel **G**–**I,** * indicates that DCs are arranged in four rows in the apical and middle turn of the P1 group. (**J**–**L**) Representative images of DCs and Cx30 expression patterns of different turns in the P3 group. (**j**–**l**) Magnified images of yellow boxes in panel **J**–**L,** * indicates that DCs are arranged in three rows in the apical and middle turn of the P3 group. (**M**) Quantifications of the number of Cx30 + cells at specific cochlear locations in different groups at P18. (**N**) Comparison of the distance between the foot of IPC and OPC in different groups. ns: not significant. Data are presented as means ± SD, ** *p* < 0.01, *** *p* < 0.001. Scale bar: 40 μm (**A**).

Next, we explored the effects of excess T3 administration at different time points after birth on the development of the OC. We labeled DCs with Cx30, a protein subunit that constitutes gap junctions, which serves as a functional marker of DCs. In the control group, Cx30 signals (green) were evenly distributed along the boundaries of all DCs (Figure 6A–C). In contrast in the P0 group, we observed that the Deiter-like cells also expressed Cx30, which suggested that these cells might have some of the functions of DCs (Figure 6D,d,E,e). When T3 was given at P1, we also observed four rows of Cx30-expressing DCs in the apical and middle turns (Figure 6G,g,H,h). However, T3 given at P3 did not significantly affect the number of DCs (Figure 6J–L,j–l). Quantitative results showed that the number of DCs was significantly increased in apical and middle turns from the P0 and P1 groups (*n* = 4, *p* < 0.01) (Figure 6M). The distance between the feet of the IPCs and OPCs was also reduced in the apical and middle turns of the P0 and P1 groups (Figure 6N). These parameters did not change significantly in the P3 group. Our results reveal that excess T3 regulates the development of the OC, especially for DCs, in a narrow postnatal time window.

### 2.5. Ultrastructural Changes of SCs in T3-Treated Mice

**Figure 7:**
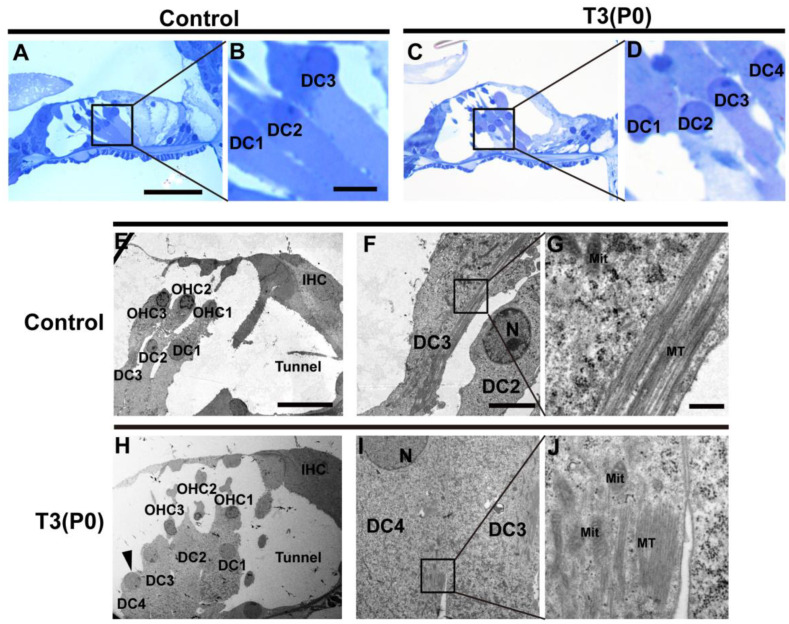
Ultrastructural of DCs were observed in control or T3 group. (**A**,**C**) The morphology of the OC in the apical of the control group and the P0 group at P18. (**B**,**D**) Magnified images of black boxes in the panel **A** and **C**. (**E**) The ultrastructure of organ of Corti in apical turns from the control group. (**F**,**G**) Magnified images show the ultrastructural of DCs in the control group. (**H**) The ultrastructure of OC in apical turns from the P0 group. The black arrowhead in panel J indicates the extra DCs (DC4). (**I**,**J**) Magnified images show the ultrastructural of DC4 in the T3 treatment group. The scales in the panel **A**, **B**, **E**, **F**, and **G** represent 40, 5, 20, 5, and 0.5 μm, respectively. N: nucleus, MT: microtubule, Mit: mitochondria.

Radial sections of the cochlea revealed the nuclei of three rows of DCs in the control group (Figure 7A,B). However, in the apical turn of the P0 group, we observed nuclei of four rows of DCs (Figure 7C,D). In addition, ultrastructural examination showed the presence of three rows of DC cell bodies in the control group and bundles of microtubules and normal mitochondria in DCs (Figure 7E–G). In the P0 group, we observed four rows of DC cell bodies (Figure 7H). The phalangeal processes of extra DCs showed normal architecture of the bundles of microtubules and mitochondria (Figure 7I,J), which indicated that the overproduced Deiter-like cells have a similar structure to normal DCs and could potentially function similarly.

### 2.6. Characterization of Gene Expression Changes in the Cochleae of T3-Treated Mice by Real-Time qPCR

**Figure 8:**
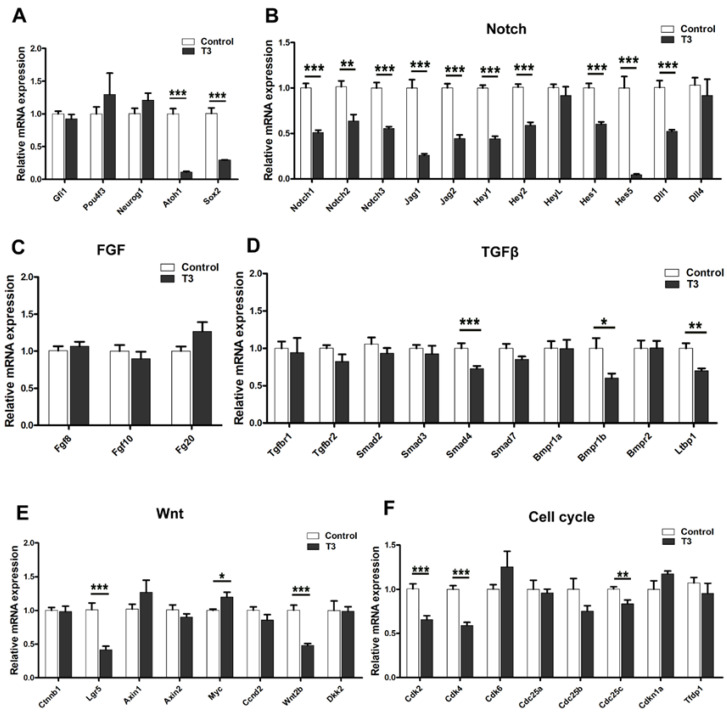
Characterization of gene expression changes in the cochlea of T3 treated mice. (**A**–**F**) Relative mRNA expression levels of genes related to SCs development (**A**), Notch signaling (**B**), FGF signaling (**C**), TGFβ signaling (**D**), Wnt signaling (**E**), and cell cycle pathways (**F**). Data are presented as means ± SD, * *p* < 0.05, ** *p* < 0.01, *** *p* < 0.001.(*n* = 6/group).

To investigate the mechanism involved in the T3-induced remodeling of the OC, we performed qPCR to analyze the expression levels of a series of genes regulating the development of the inner ear. Neonatal mice were injected with T3 at P0 andP1 and then sacrificed at P4 for qPCR to analyze. Mice without T3 treatment save as control group, the mRNA expression of Atoh1 and Sox2, two transcription factors that regulate the development of HCs and SCs, was significantly downregulated (Figure 8A). However, the other important factors Pou4f3, Neurog1, and Gfi1 did not change significantly. In addition, we analyzed the Notch, Wnt, TGFβ, and FGF signaling pathways as well as cell cycle signaling pathways and found that the transcription levels of Notch pathway-related genes, such as Notch1, Notch2, Notch2, Notch3, Jag1, Jag2, Hey1, Hey2, Hes1, Hes5, and Dll1 were significantly downregulated (Figure 8B). In contrast, expression of FGF and most TGFβ signaling pathway genes did not change significantly, while only Smad4, Bmpr1b, and Ltbp1 were downregulated (Figure 8C,D). In the Wnt pathway, the mRNA expression levels of Lgr5 and Wnt2b were significantly downregulated and other related genes were not significantly changed (Figure 8E). In addition, we found that the cell cycle-dependent kinases Cdk2 and Cdk4, and cell division cyclin Cdc25c, were downregulated in cochleae of T3-treated mice (Figure 8F). All these results suggest that T3 may lead to the overproduction of DCs mainly through downregulation of the Notch signaling pathway in early cochlear development. 

### 2.7. Effects of Excess T3 Combined with Regulated Sox2 on the Remodeling of OC

**Figure 9:**
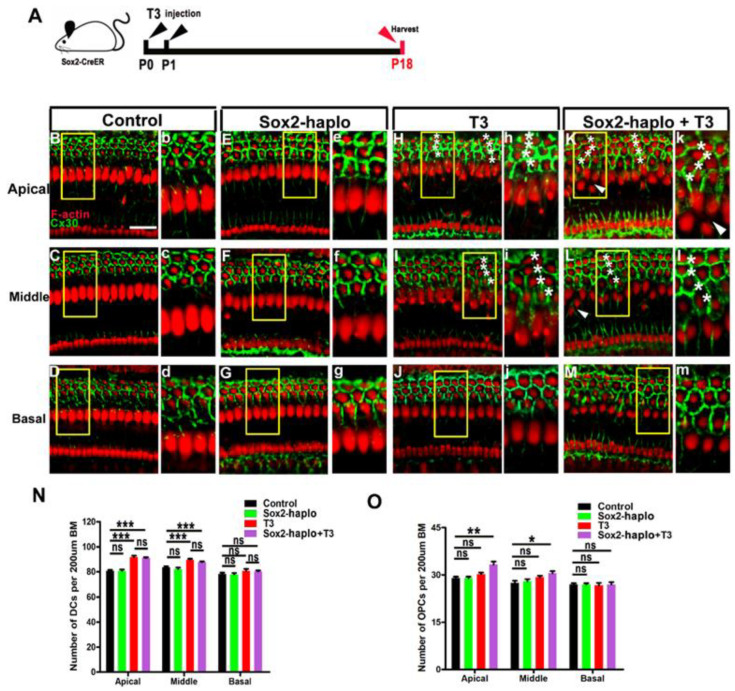
Effects of T3 combined with downregulating Sox2 on the remodeling of OC**.** (**A**) Sox2 haploinsufficient mice were injected with T3 at P0 and P1, and sacrificed at P18. (**B**–**D**) F-actin (red) and Cx30 immunolabeling (green) in different turns of the control group. (**E**–**G**) Representative images of DCs and Cx30 expression patterns of different turns of the Sox2 haplo group. (**b**–**g**) Magnified images of yellow boxes in panel B–G. (**H**–**J**) Representative images of DCs and Cx30 expression patterns of apical, middle, and basal turns of alone were treated with the T3 group, respectively. (**h**–**j**) Magnified images of yellow boxes in panel H–J.* indicates DCs were arranged in four rows in apical and middle turns of T3 alone treated group (**H**,**I**, **h**,**i**). (**K**–**M**) Representative images of DCs and Cx30 expression patterns in different turns of the Sox2 haplo + T3 group. (**k**–**m**) Magnified images of yellow boxes in panel K–M, * indicates DCs were arranged in four rows in apical and middle turns (**K**,**L**,**k**,**l**), and white arrowheads indicate the regions where OPCs were arranged in two rows in apical and middle turns (**K**,**L**). (**N**) Comparison of the number of DCs at specific cochlear locations in the different groups. (**O**) Comparison of the number of OPCs at specific cochlear locations in the different groups. Data are presented as means ± SD, ns: not significant, *** *p* < 0.001, ** *p* < 0.01, * *p* < 0.05. Scale bar: 40 μm (**B**).

**Figure 10:**
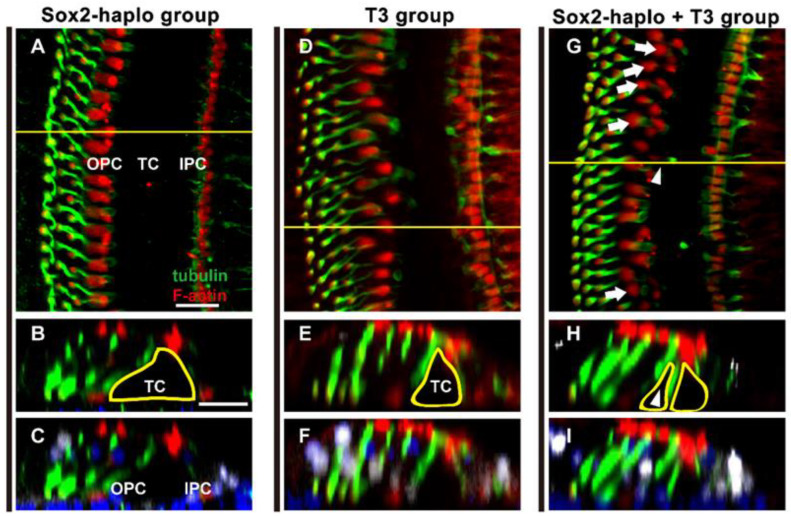
The combination of T3 and Sox2 haploinsufficiency induced the remodeling of the tunnel of Corti. (**A**–**F**) F-actin (red) and acetylated α-tubulin immunolabeling (green) in apical of the Sox2 help or T3 group, and cross-sectional views were generated to show the morphology of the OC. (**G**–**I**) F-actin and acetylated α-tubulin immunolabeling in the apical turn of the Sox2 haplo + T3 group, and cross-sectional views were generated to show the morphology of the OC. The yellow line indicate the location of the cross-section, white arrows indicate the regions where OPCs were arranged in two rows, white arrowheads indicate the newly formed tunnels of Corti. Scale bar: 40 μm (**A**,**B**).

Recent studies have shown that Sox2^CreER/+^ mice exhibit Sox2 haploid insufficiency due to one of the alleles being replaced by CreER [26]. Using this characteristic, Sox2^CreER/+^ mice were injected with T3 to explore the effect of T3 combined with Sox2 downregulation on the development of SCs in the inner ear (Figure 9A). In Sox2 haploinsufficient (Sox2 haplo) mice, three rows of DCs were neatly arranged, and Cx30 was observed at the edge of all DCs—same as in the control group (Figure 9B–G,b–g). In the T3 and the Sox2 haplo + T3 groups, four rows of DCs were observed in the apical and middle turns (Figure 9H,I,K,L), and the quantified results showed no significant difference in the number of DCs between the T3 and the Sox2 haplo + T3 groups (Figure 9N). However, ectopic OPCs were observed in the apical and middle turns of the Sox2 haplo + T3 group (Figure 9K,k,L,l). Statistical analysis showed that the number of OPCs was significantly increased in the apical and middle turns of the Sox2 haplo + T3 group (n = 4, *p* < 0.01) (Figure 9O). These results suggest that T3 combined with Sox2 downregulation did not aggravate the overproduction of DCs induced by T3, but did induce the overproduction of OPCs (white arrows, Figure 10G). Moreover, extra OPCs in the Sox2 haplo + T3 group appeared to form new tunnels of Corti that affected the structure of the OC (white arrowhead, Figure 10G,H). The yellow lines show the boundaries of the tunnel of Corti (Figure 10B,E,H).

## 3. Discussion

### 3.1. T3 Is an Exogenous Factor Involved in Stereocilia Formation by Cochlear Hair Cells 

Over recent decades, a series of studies have focused on the role of thyroid hormones in fetal tissue differentiation and development [27]. Fetal nervous system development is highly sensitive to thyroid hormones, and maternal thyroid hormone disorder can cause fetal central nervous system symptoms including hearing, speech, and color vision impairments, and squint [28,29]. A previous study showed that injections of T3 resulted in expected increases in serum T3 concentrations, with daily injections of 0.01, 0.1, and 2.0 µg T3 up to P5 resulting in serum T3 levels that were increased approximately 12-, 80-, and 280-fold, respectively [30]. T3-induced hearing loss in mice was concentration-dependent, with a dose of 0.1 µg T3/d resulting in a threshold of approximately 70 dB SPL, whereas 1.5 or 2.0 µg T3/d resulted in thresholds of more than or equal to 90 dB SPL [30]. A single injection of T3 at P0 resulted in significant hearing loss, whereas T3 given at P3 or later did not significantly change thresholds compared to saline-treated groups [18]. In this study, our results indicated that administration of excess T3 in the early postnatal period (P0 or P1) induced severe hearing loss in mice (Figure 1). However, we did not observe significant degeneration of HCs, suggesting that the cause of hearing loss is not simply HC death. When T3 was given at P3 (the P3 group), mice exhibited normal hearing at P18. Observations by us and others have proven that P0–P2 is the critical period when deafness is caused by excess T3 [18]. In addition, caspase-3+ cells were detected in the GER of the P0 group at P6, while apoptosis of the GER in the control group was not triggered at this time. However, a recent study by Borse et al. reported that macrophages were recruited into the GER region after initiation of GER regression during cochlear remodeling [17]. In this study, apoptotic signals in the GER region were detected at P5. This difference in timing may be due to differences in experimental technique and mouse strain used. Excess T3 advances the overall program of apoptosis, with regression of the GER initiated at P3 and creating a large cavity known as the inner spiral sulcus at P5 in the T3 treatment group, whereas in normal mice this process occurs at P7 [18]. Premature degeneration of the GER triggered by T3 resulted in the advanced opening of the tunnel of Corti. However, it remains unclear whether pre-maturation of the OC is directly related to hearing loss.

Stereocilia are mechanical sensors located in the cochlear sensory cells that convert sound stimuli into electrical signals, and normal auditory function depends on the organization and morphology of the stereocilia, thus they are thought to be critical for mammalian hearing and balance [31,32]. Disorders of the stereocilia hair bundle structure are involved in various forms of congenital or progressive hearing loss [33,34,35]. We observed that administration of excess T3 in the early postnatal period (P0 or P1) caused a disturbance in the arrangement of the stereocilia of HCs, while the stereocilia showed normal structure in the P3 group. As shown in the picture (Figure 3), the stereocilia hair bundles were disordered and varied in length in the T3-treated group. Similar to the effect on hearing, only P0–P2 administration of excess T3 resulted in abnormal stereocilia development. In addition, we observed the reduced function of MET channels located at the apical junction of hair cell stereocilia in the P0 group (Figure 4A,B). Additionally, lower DPOAE levels were also found in the P0 group (Figure 4C,D). This evidence indicated that the OHC abilities of mechanoelectrical transduction and amplification were both significantly impaired. Combined with analysis of the audiological phenotype and pathological phenotype, the results suggested that the disturbance of HC stereocilia and impaired function of OHCs were the main causes of hearing loss caused in the P0 and P1 groups. Based on the above results, excess T3 during the very early stage after birth does not affect HC survival but does cause dysfunction of HCs with abnormal development of stereocilia, which would be a novel mechanism of thyroid hormone-induced hearing loss.

### 3.2. T3 Regulates the Production of SCs during Critical Periods of Cochlear Development 

TRs, deiodinase, and thyroid hormone transporter are widely expressed in the cochlea [30,36], which suggests that cochleae are the targets of thyroid hormone regulation of inner ear development. Forrest et al. reported that T3 regulates cochlear remodeling, which involves premature regression of the GER [18]. However, the effect of T3 on the remodeling of the OC during early development has not been further explored. Here, we observed that treatment with T3 in the early development stage (P0 or P1) resulted in the overproduction of DCs. Immunostaining results showed that these cells were connected with adjacent DCs by gap junctions. Microtubules, labeled by acetylated α-tubulin, were found in the body and phalangeal processes of the cells. This indicated that these Deiter-like cells may be functioning normally and can communicate intercellularly with adjacent SCs. We further speculate that they may be used as the reserve of DCs which can support new regenerated OHCs. Previous studies mainly focused on regulating SC proliferation by regulating proliferation-related genes. Our study showed that endocrine signals also contribute to the regulation of SC proliferation and development. In addition, T3 administration at P3 did not affect the number of DCs, suggesting that there was a narrow time window during which T3 regulated the proliferation of SCs.

In adult mammals, damage to sensory cells in the inner ear causes permanent hearing loss because degeneration of HCs is irreversible, whereas HCs can spontaneously regenerate from supporting cells (SCs) after injury in birds and fish [37]. Recent studies have shown that HCs can also be regenerated from SCs in newborn mice [38,39,40], however this spontaneous regenerative ability rapidly diminishes with age. Current research suggests that there are two mechanisms for HC regeneration in mammals, one of which is the direct trans-differentiation of SCs into new HCs [41]. The second mechanism involves the SCs or progenitor cells of the inner ear proliferating and then differentiating into new HCs [42,43,44]. The common feature of both pathways is that the new HCs are derived from SCs, which suggests that SCs in the inner ear are the key factor necessary for HC regeneration. Therefore, increasing the number of SCs is an important step in achieving HC regeneration. Multiple signaling pathways (Wnt, Notch, FGF, IGF, and Shh) are involved in the development and proliferation of SCs, and regulation of related genes leads to an increase in the number of SCs in the inner ear [45,46,47]. Our results reveal that endocrine signals regulate the proliferation of inner ear SCs during critical periods of cochlear development, providing a reference to coordinate the multi-factor regulation of SC proliferation and HC regeneration.

Recent studies have shown that multiple signaling pathways are involved in regulating the development of HCs and SCs, among which the Notch signaling pathway plays an important role in this process [48]. In the sensory epithelium of the inner ear, HCs express the Notch ligands Dll1, Dll3, Dll4, Jag1, and Jag2, while SCs express Notch downstream genes including Hes1, Hes5, Hey1, Hey2, and Heyl. Notch-mediated lateral inhibition maintains SCs in a quiescent state, and suppression of Notch signaling by drugs or genetic ablation of Notch effector genes leads to excessive formation of HCs or overproduction of SCs [49,50]. Hes1, Hes5, and Hey1 are three of the important Notch downstream transcription factors, and knock-out of Hes1, Hes5, and Hey1 in the inner ear results in extra HCs accompanied by overproduction of SCs [46,51,52]. In our study, real-time quantitative PCR results showed that the expression of genes related to the Notch signaling pathway including Hes1, Hes5, and Hey1 was significantly downregulated. Therefore, the T3-induced overproduction of SCs may be directly related to notch downregulation. We did not find any expression changes of FGF signaling pathways, which suggests that the phenotype of overproduction of SCs induced by T3 might not involve FGF pathways. In addition, the expression of individual genes in some classical pathways that regulate DC proliferation was significantly altered, such as Smad4, Bmpr1b, Ltbp1 Lgr5, and Wnt2b which were significantly downregulated, but the significance of these gene changes remains unclear. In the future, the effects of T3 on more pathways related to inner ear development remain to be explored. 

### 3.3. The Combination of T3 and Sox2 Haploinsufficiency Regulates Not Only the Number of SCs but Also the Fine Structure of the OC

Expression of the transcription factor Sox2 was significantly downregulated in the T3 group. Based on the characteristics of Sox2^CreER/+^ mice with Sox2 haploinsufficiency [26], we constructed a mouse model of T3 combined with downregulation of Sox2 by giving T3 to Sox2^CreER/+^ mice. The results showed that excess T3 treatment combined with downregulation of Sox2 not only resulted in overproduction of DCs but also led to a large number of OPCs. In cochlear development, the formation of the tunnel of Corti is a milestone in OC maturation. These ectopic OPCs resulted in a great change in the morphology of the normal OC. This phenomenon indicates that T3 combined with local transcription factors (such as Sox2) may regulate and even induce the formation of the complex spatial structure of the OC. The key signaling pathway mediating SC proliferation and remodeling of the fine structure of the OC induced by T3 still needs further study. The idea that endocrine signaling may combine with gene programming to regulate cochlear development and SC proliferation is a novel proposal that will open up new aspects of the field of study and potentially lead to the development of new therapies.

In general, our results show that excess T3 given at an early stage (P0 or P1) leads to severe hearing loss with abnormal stereocilia alignment and HC mechanosensory dysfunction. However, the molecular mechanism by which T3 induces the abnormal development of the stereocilia of OHCs remains unclear. The key molecules of T3 leading to abnormal stereocilia of OHCs need to be further explored. Moreover, overproduction of Deiter-like cells and a series of genes related to the development of cochlear sensory epithelium were significantly downregulated in the T3 group, but the significance of these gene changes remains unclear.

## 4. Materials and Methods

### 4.1. Mouse Models

C57BL/6J mice were approved by the Committee of Tongji Medical College, Huazhong University of Science and Technology. Neonatal mouse pups were subcutaneously injected with 2.0 µg of T3 (T2877, Merck KGaA, Darmstadt, Germany) in a volume of 10 µL, or the equivalent volume of saline, at P0 (the P0 group), P1 (the P1 group), or P3 (the P3 group). The concentration and total dose of T3 were based on previous studies and combined with data from our preliminary experiment; mice at this dose show an obvious audiological phenotype without causing gross developmental abnormalities [30]. Both female and male neonatal mouse were included in the experiment and randomly grouped. Mice without T3 treatment were saved as a control group (*n* = 4 in each group). The preparation of T3 was performed strictly in accordance with the manufacturer’s instructions. Operators wore masks and gloves for self-protection, and waste disposal was standardized. All mice were raised in the specific-pathogen free (SPF) Experimental Animal Center and housed at 22 ± 1 °C under a standard 12 h light/dark cycle and were allowed free access to water and a regular mouse diet.

The Sox2 haploinsufficient mice were a gift from Prof. Zhang at Southeast University in China. This line (Sox2^CreER/+^) was generated as an inserted targeted mutation in the single exon of the Sox2 gene, resulting in Sox2 haploinsufficiency [26,53]. Details of this line are given in the study by Zhang et al. [41]. Sox2 haploinsufficient mice treated with T3 at P0 (the Sox2 haplo+T3 group) were used to investigate their combined effects on cochlear development.

The genotyping primers for Sox2^CreER/+^mice were as follows:

wild type (F) 5′-CTAGGCCACAGAATTGAAAGATCT-3′; 

wild type (R) 5′-GTAGGTGGAAATTCTAGCATCATCC-3′;

mutant (F) 5′-GCG GTCTGGCAGTAAAAACTATC-3′;

mutant (R) 5′-GTGAAACAGCAT TGCTGTCACTT-3′. 

### 4.2. Auditory Brainstem Response (ABR) and Distortion Product Otoacoustic Emission (DPOAE)

The auditory thresholds of different groups were determined by ABR detection at P18 (*n* = 4 mice in each group). The details of the ABR test were as described in our previous study [54]. Briefly, the mice were deeply anesthetized and three subcutaneous electrodes were placed at the vertex of the skull, the tested ear, and the contralateral ear. Click and tone burst stimuli at frequencies of 8, 16, 24, 32, and 40 kHz were generated. The responses were recorded and determined by decreasing sound intensities from 90 dB in 10 dB steps, which narrowed to 5 dB steps when near the threshold. The lowest sound intensity that could be recognized was determined to be the auditory threshold. DPOAE was measured at P20 (*n* = 5 mice in each group). The details of the DPOAE test were as described in our previous study [55].

### 4.3. Immunofluorescence

For activated caspase-3 immunostaining, mice were anesthetized and sacrificed at P6 or P11. For counting cochlear HCs and DCs, mice (*n* = 4 in each group) were sacrificed at P18. The cochleae were carefully dissected in 0.01 M PBS and then fixed in 4% paraformaldehyde. For flattened cochlear preparations, the samples were rinsed three times with PBS and decalcified with 10% disodium EDTA at 4 °C for two days. Each stretched cochlear preparation was carefully dissected and incubated in blocking solution at room temperature for 1 h, then incubated with polyclonal rabbit anti-myosin 7a antibody (1:500 dilution, 25–6790, Proteus Bio-Sciences, Ramona, CA, USA), polyclonal goat anti-Sox2 antibodies (1:100 dilution, sc-17320, Santa Cruz Biotechnology, Santa Cruz, CA, USA), monoclonal rabbit anti-α-tubulin antibody (1:200 dilution, ab179484, Abcam, Cambridge, UK), or polyclonal rabbit anti-Cx30 antibodies (1:200 dilution, 40–7400, Invitrogen, Carlsbad, CA, USA). After washing with PBST three times, the samples were incubated with fluorescent secondary antibodies (1:200 dilution, ANT032, Antgene, Wuhan, China) for 2 h in the dark. Phalloidin (P5282, Sigma, St. Louis, MO, USA) was used for fluorescent visualization of HC F-actin, and nuclei were labeled with DAPI (C1005, Beyotime Biotechnology, Shanghai, China). All images were scanned with a laser scanning confocal microscope (Nikon, Tokyo, Japan). The distance between OPCs and IPCs was measured using Image J software (Version 1.48, National Institutes of Health, Bethesda, MD, USA). For outer hair cell and DCs counting, cells were counted from 60 X images taken from the apex, middle, and base of the basilar membrane.

### 4.4. Resin Sections and Transmission Electron Microscopy (TEM)

The detailed methods for TEM have been described previously [55]. Briefly, mice were anesthetized and sacrificed at P18 (*n* = 4 mice in each group). After decalcification with 10% disodium EDTA for 48 h, each sample was then immersed in 1% osmium tetroxide to post-fix for 1 h. Samples were dehydrated through a graded ethanol series, before embedding in resin. Sections (1.5 μm in thickness) were stained with toluidine blue (89640-5G, Sigma-Aldrich, St. Louis, MO, USA) for observation, and ultrathin sections were stained with uranyl acetate and lead citrate and examined by TEM. 

### 4.5. Scanning Electron Microscopy (SEM)

The morphology of HC stereocilia was observed by SEM at P18 (*n* = 4 mice in each group). As previously described [54], after fixation and decalcification, the cochleae were carefully dissected to expose the basilar membrane. Then, the samples were dehydrated in increasing ethanol concentrations, dried (HCP-2, Critical Point Dryer, HITACHI, Tokyo, Japan), and sputter-coated with a layer of gold (Eiko Engineering, Tokyo, Japan). Stereocilia bundles were observed in the three turns of the cochlea. Images were captured using a scanning electron microscope (VEGA 3 LMU, Tescan, Brno, Czech Republic).

### 4.6. FM1-43 Imaging

FM1-43 loading of HCs was used to assess the function of mechano-transduction channels. Mice (*n* = 4 in each group) were sacrificed at P18, and cochleae were quickly dissected from the temporal bones. The samples were incubated in a culture loaded with 4 µM FM1-43 (T35356, Invitrogen) for 30 s, and then fixed in 4% paraformaldehyde for 1 h. Samples were washed with 0.01 M PBS three times before imaging with a confocal microscope; all operations were performed at room temperature. DAPI was used for nuclear staining. Next, 40 OHCs were selected from four mice in each group for fluorescence quantification. The intake of FM1-43 was determined by mean fluorescence intensity.

### 4.7. Real-Time Quantitative Polymerase Chain Reaction (RT-qPCR)

Neonatal mice were injected with T3 at P0 and P1, then sacrificed at P4. The cochleae were removed and dissected in cold Hanks’ balanced salt solution (H1045, Solarbio, beijing, China). The membranous cochlear duct sourced from one cochlea was used to generate one sample. The detailed methods for the RNA extraction and reverse transcription were as described previously [56]. Total RNA was extracted from the collected tissues using an RNAprep Pure Tissue Kit (Tiangen Biotech Co., Ltd., Beijing, China) and was reverse transcribed using a PrimeScript RT Reagent Kit with gDNA eraser (Takara Bio Inc. Shiga, Japan). RT-qPCR was performed in a Roche LightCycler 480 instrument (Roche Diagnostics Ltd., Basel, Switzerland). Real-time qPCR conditions were an initial denaturing step of 15 s at 95 °C followed by 40 cycles of 15 s denaturation at 95 °C, 60 s annealing at 60 °C, and 20 s extension at 72 °C. The transcriptional expression was normalized to the expression of GAPDH and the relative expression level between the control and T3 group was calculated using the 2^−∆∆CT^ method [41]. The real-time qPCR primers are shown in Supplementary Table S1.

### 4.8. Statistical Analysis 

Data are presented as means ± SD, statistical analyses were conducted using GraphPad Prism (Version 8.0, GraphPad Software Inc., La Jolla, CA, USA) and SPSS software (version 19, IBM SPSS Statistics, IBM Corp., Armonk, NY, USA). One-way ANOVA followed by a Dunnett multiple comparisons test was used when there was only one factor. Two-way-ANOVA multiple comparisons test was used when two factors were involved. *p* < 0.05 was considered to be statistically significant.

## 5. Conclusions

Our results suggest that (I) T3 is an exogenous factor involved in mouse stereocilia formation and HC functions; (II) T3 regulates the production of mouse SCs during critical periods of cochlear development; and (III) the combination of T3 and Sox2 haploinsufficiency regulates not only the number of SCs but also the structure of the OC. Our findings provide new evidence for the role of endocrine signaling in regulating the development of mouse cochlear sensory epithelium.

## Data Availability

The original data supporting the conclusions of this article will be made available by the authors, further inquiries can be directed to the corresponding author.

## Supplementary Materials

The following supporting information can be downloaded at: https://www.mdpi.com/article/10.3390/ijms24054559/s1.
