# Supplementary material for: The Dual Roles of Triiodothyronine in Regulating the Morphology of Hair Cells and Supporting Cells during Critical Periods of Mouse Cochlear Development
Source: Int J Mol Sci. 2023 Feb 25;24(5):4559. doi: 10.3390/ijms24054559 (PMC10003541; doi:10.3390/ijms24054559)
Supplement: Supplementary file 1 [file ijms-24-04559-s001.zip › ijms-2164303-supplementary.pdf]

Table S1. The primers for real-time qPCR were as follows:

Gene Name	Forward (5'–3')	Reverse (5'–3')
GAPDH	GAAGGTCGGTGTGAACGGAT	CTCGCTCCTGGAAGATGGTG
Gfi1	AGAAGGCGCACAGCTATCAC	GGCTCCATTTTCGACTCGC
Pou4f3	CGACGCCACCTACCATACC	CCCTGATGTACCGCGTGAT
Neurog1	CCAGCGACACTGAGTCCTG	CGGGCCATAGGTGAAGTCTT
Atoh1	ATGCACGGGCTGAACCA	TCGTTGTTGAAGGACGGGATA
Sox2	CTGTTTTTTCATCCCAATTGCA	CGGAGATCTGGCGGAGAATA
Notch1	CCGTGTAAGAATGCTGGAACG	AGCGACAGATGTATGAAGACTCA
Notch2	ATGTGGACGAGTGTCTGTTGC	GGAAGCATAGGCACAGTCATC
Notch3	TGCCAGAGTTCAGTGGTGG	CACAGGCAAATCGGCCATC
Jag1	ATGCAGAACGTGAATGGAGAG	GCGGGACTGATACTCCTTGAG
Jag2	CAATGACACCACTCCAGATGAG	GGCCAAAGAAGTCGTTGCG
Hey1	CCGACGAGACCGAATCAATAAC	TCAGGTGATCCACAGTCATCTG
Hey2	AAGCGCCCTTGTGAGGAAA	TCGCTCCCCACGTCGAT
HeyL	GCGCAGAGGGATCATAGAGAA	TCGCAATTCAGAAAGGCTACTG
Hes1	GCTTCAGCGAGTGCATGAAC	CGGTGTTAACGCCCTCACA
Hes5	GGCGGTGGAGATGCTCAGT	GCTGCTCTATGCTGCTGTTGA
Dll1	CAGGACCTTCTTTCGCGTATG	AAGGGGAATCGGATGGGGTT
Dll4	TTCCAGGCAACCTTCTCCGA	ACTGCCGCTATTCTTGTCCC
Fgf8	AGAGCCTGGTGACGGATCA	CTTCCAAAAGTATCGGTCTCCAC
Fgf10	TCAGCGGGACCAAGAATGAAG	CGGCAACAACTCCGATTTCC
Fgf20	AGGATCACAGTCTCTTCGGTATC	GTCATTCATCCCAAGGTACAGG
Tgfbr1	TCCCAACTACAGGACCTTTTTCA	GCAGTGGTAAACCTGATCCAGA
Tgfbr2	CCGCTGCATATCGTCCTGTG	AGTGGATGGATGGTCCTATTACA
Smad2	ATGTCGTCCATCTTGCCATTC	AACCGTCCTGTTTTCTTTAGCTT
Smad3	CACGCAGAACGTGAACACC	GGCAGTAGATAACGTGAGGGA
Smad4	ACACCAACAAGTAACGATGCC	GCAAAGGTTTCACTTTCCCCA
Smad7	GGCCGGATCTCAGGCATTC	TTGGGTATCTGGAGTAAGGAGG
Bmpr1a	AACAGCGATGAATGTCTTCGAG	GTCTGGAGGCTGGATTATGGG
Bmpr1b	CCCTCGGCCCAAGATCCTA	CAACAGGCATTCCAGAGTCATC
Bmpr2	TTGGGATAGGTGAGAGTCGAAT	TGTTTCACAAGATTGATGTCCCC
Ltbp1	CCAGTCCCAAGTCTCTTACCA	CTGGAAGCATCGGCCAAGT
Ctnnb1	ATGGAGCCGGACAGAAAAGC	CTTGCCACTCAGGGAAGGA
Lgr5	TCTTCACCTCCTACCTGGACCT	GGCGTAGTCTGCTATGTGGTGT
Axin1	CTCCAAGCAGAGGACAAAATCA	GGATGGGTTCCCCACAGAAATA
Axin2	TGACTCTCCTTCCAGATCCCA	TGCCCACACTAGGCTGACA
Myc	ATGCCCCTCAACGTGAACTTC	GTCGCAGATGAAATAGGGCTG
Ccnd2	GAGTGGGAACTGGTAGTGTTG	CGCACAGAGCGATGAAGGT
Wnt2b	GACACGTCCTGGTGGTACATA	GTCTGGGTAGCGTTGACACA
Dkk2	ACCCGCTGCAATAATGGAATC	ATGGTTGCGATCTCTATGCCG
Cdk2	CCTGCTTATCAATGCAGAGGG	GTGCTGGGTACACACTAGGTG
Cdk4	ATGGCTGCCACTCGATATGAA	TGCTCCTCCATTAGGAACTCTC
Cdk6	TCTCACAGAGTAGTGCATCGT	CGAGGTAAGGGCCATCTGAAAA
Cdc25a	ACAGCAGTCTACAGAGAATGGG	GATGAGGTGAAAGGTGTCTTGG
Cdc25b	TCCGATCCTTACCAGTGAGG	GGTCTCTGGAAGCGCACATT
Cdc25c	ATGTCTACAGGACCTATCCCAC	ACCTAAAACTGGGTGCTGAAAC
Cdkn1a	CCTGGTGATGTCCGACCTG	CCATGAGCGCATCGCAATC
Tfdp1	TTGAAGCCAACGGAGAACTAAAG	TGGACTGTCCGAAGGTTTTTG
